# Genome-Wide Identification and Characterisation of Cytokinin-O-Glucosyltransferase (CGT) Genes of Rice Specific to Potential Pathogens

**DOI:** 10.3390/plants11070917

**Published:** 2022-03-29

**Authors:** Wadzani Palnam Dauda, Veerubommu Shanmugam, Aditya Tyagi, Amolkumar U. Solanke, Vishesh Kumar, Subbaiyan Gopala Krishnan, Bishnu Maya Bashyal, Rashmi Aggarwal

**Affiliations:** 1 ICAR-Indian Agricultural Research Institute, New Delhi 110012, India; wadzani_11364@iari.res.in (W.P.D.); adityatyagi1993@gmail.com (A.T.); gopal.s@icar.gov.in (S.G.K.); bishnumayabashyal@gmail.com (B.M.B.); rashmi.aggarwal2@gmail.com (R.A.); 2 Crop Science Unit, Department of Agronomy, Federal University, Gashua 1005, Nigeria; 3 ICAR-National Institute for Plant Biotechnology, New Delhi 110012, India; amol.solanke@icar.gov.in (A.U.S.); visheshkumar08@gmail.com (V.K.)

**Keywords:** rice, cytokinin glycosyltransferases (CGTs), sheath blight (ShB), blast, bacterial leaf blight (BLB), family 1 glycosyltransferases (GT1s), plant secondary product glycosyltransferases (PSPG)

## Abstract

Cytokinin glucosyltransferases (CGTs) are key enzymes of plants for regulating the level and function of cytokinins. In a genomic identification of rice CGTs, 41 genes with the plant secondary product glycosyltransferases (PSPG) motif of 44-amino-acid consensus sequence characteristic of plant uridine diphosphate (UDP)-glycosyltransferases (UGTs) were identified. In-silico physicochemical characterisation revealed that, though the CGTs belong to the same subfamily, they display varying molecular weights, ranging from 19.6 kDa to 59.7 kDa. The proteins were primarily acidic (87.8%) and hydrophilic (58.6%) and were observed to be distributed in the plastids (16), plasma membrane (13), mitochondria (5), and cytosol (4). Phylogenetic analysis of the CGTs revealed that their evolutionary relatedness ranged from 70–100%, and they aligned themselves into two major clusters. In a comprehensive analysis of the available transcriptomics data of rice samples representing different growth stages only the CGT, *Os04g25440.1* was significantly expressed at the vegetative stage, whereas 16 other genes were highly expressed only at the reproductive growth stage. On the contrary, six genes, *LOC_Os07g30610.1, LOC_Os04g25440.1, LOC_Os07g30620.1, LOC_Os04g25490.1, LOC_Os04g37820.1,* and *LOC_Os04g25800.1,* were significantly upregulated in rice plants inoculated with *Rhizoctonia solani* (RS), Xoo (*Xanthomonas oryzae* pv. *oryzae*) and Mor (*Magnaporthe oryzae*). In a qRT-PCR analysis of rice sheath tissue susceptible to *Rhizoctonia solani*, Mor, and Xoo pathogens, compared to the sterile distilled water control, at 24 h post-infection only two genes displayed significant upregulation in response to all the three pathogens: *LOC_Os07g30620.1* and *LOC_Os04g25820.1*. On the other hand, the expression of genes *LOC_Os07g30610.1, LOC_Os04g25440, LOC_Os04g25490*, and *LOC_Os04g25800* were observed to be pathogen-specific. These genes were identified as the candidate-responsive CGT genes and could serve as potential susceptibility genes for facilitating pathogen infection.

## 1. Introduction

Rice is one of the most widely cultivated crops globally, supplying up to 50% of dietary calories, mainly in Asian and African countries [1]. To meet the ever-increasing food demand for the projected human population of 9 billion by 2030, global rice production would have to increase by 40% over the present-day production [2]. However, increasing yield to meet the expected global food demand is greatly constrained by fungal and bacterial diseases affecting the crop [3]. Sheath blight (ShB) and the blast caused by *Rhizoctonia solani*, RS Kühn (teleomorph: *Thanatephorus cucumeris* (A.B. Frank) Donk) and *Magnaporthe oryzae, Mor* (teleomorph: *Pyricularia oryzae*) (Herbert) Barr, respectively, are two major fungal diseases, and bacterial leaf blight (BLB), caused by the bacterium *Xanthomonas oryzae* pv. *oryzae, Xoo* is amongst the top 10 economically important bacterial diseases limiting rice production globally [4]. Understanding their interactions with the rice host during pathogenesis may enable the development of effective strategies to contain them.

In pathogen–host interactions, the recognition of conserved pathogen-associated molecular patterns (PAMPs) or the damage-associated molecular patterns (DAMPs) of the pathogenic microbes by pattern-recognition receptors (PRRs) of the host plasma membrane triggers basal resistance or PAMP-triggered immunity (PTI), the plant’s first line of defence [5]. In PTI, the defence reactions are manifested through the accumulation of reactive oxygen species (ROS), metabolites/enzymes with antimicrobial activities, and, at later stages, cell wall thickening. The pathogens employ various mechanisms to manipulate or overwhelm the basal defences of the host plants, including producing endogenous effector proteins and toxic metabolites [6] to facilitate infection in a process termed effector triggered susceptibility (ETS). The secreted effectors interact with specific host proteins to suppress them if they function in plant defence or activate them if they function as negative regulators of plant immunity or as susceptibility factors. The pathogen effectors also favour infection by manipulating hormonal homeostasis, either by targeting the pathway components of salicylic acid, jasmonic acid, and ethylene hormones involved in disease resistance or those of the hormones auxin, cytokinins, and gibberellic acid known to be involved in the plant developmental processes. Secretion of monooxygenase and chorismate mutase by the fungal pathogens *Mor* and *Ustilago maydis*, respectively, affect salicylic acid or jasmonic acid homeostasis during infection and favour virulence [7,8]. The pathogens also produce hormones or similar compounds, such as the coronatine of *Pseudomonas syringae* mimicking jasmonic acid in order to counteract salicylic acid accumulation [9] to favour infection.

Cytokinins (CKs) are small-molecule hormonal compounds derived from adenine [10] and occur naturally in plants. The hormones are known for cell division/cell differentiation [11] and regulating growth and development. Besides, they are known to confer abiotic and biotic stress tolerance [11]. Supplementation with cytokinins is shown to increase (cytokinin-induced immunity) or decrease (cytokinin-induced susceptibility) disease resistance in plants [12]. Cytokinin-induced immunity has been reviewed extensively [13]; the decrease in pathogen growth is accounted for by enhanced expression of defence genes regulating ROS homeostasis and is dependent or independent of the content and signalling of salicylic acid and methyl-jasmonic acid [13]. Contrary to cytokinin-induced immunity, cytokinin-induced susceptibility is a pathogen-driven process inducing low to moderate levels of cytokinin; either the pathogens themselves produce cytokinins directly or manipulate cytokinin signalling and/or content in the plants. Nevertheless, cytokinin production has mostly been limited to biotrophic and hemibiotrophic pathogens and has been correlated to the virulence of the tumour-forming pathogens *Ustilago* *maydis* [14], *Claviceps purpurea* [15], *Plasmodiophora brassicae* [16], and *Rhodococcus fascians* [17]. Lately, the production of CKs has also accounted for the virulence of non-tumour-forming pathogens such as Mor [18]. Several studies also indicate CKs as the pathogenicity factors of *C. purpurea* [15,18] and *U. maydis* [19]. In plants, CKs delay senescence by limiting oxidative burst and maintaining photosynthesis activity [20] and are hence activated only by hemibiotrophic pathogens in order to avoid cell death from draining of nutrients from the host cells (otherwise the dead cells would be employed in defence reactions) [13]. The activated CKs accumulate in “green islands”—the photosynthetically active tissues around the lesions caused by the hemibiotrophic pathogens [18]. Activation of CK signalling in plants through effectors has been reported in *Pseudomonas syringae* pv. Tomato [21].

The levels of cytokinins in the plants are controlled by biosynthesis, destruction, and inactivation [12]. CK synthesis is usually accomplished by the de novo synthesis [22] pathway, including the adenosine monophosphate (AMP) pathway, the ATP/ADP pathway, and the alternative iPMP-independent pathway. Few cytokinins are synthesized by the transport RNA (tRNA) pathway [14,23]. Destruction and/or inactivation of cytokinins is usually accomplished by cytokinin oxidase/dehydrogenase (CKX) and via glucosylation by cytokinin glucosyltransferases (CGTs). In plants, cytokinins primarily exist as glycosides in various forms, and glycosylation of cytokinins is catalysed by family 1 glycosyltransferases (GT1s) known as cytokinin glycosyltransferases (CGTs). The GT1s form the cytokinin glycoside product by transferring an active sugar donor, usually a UDP-glycosyl group, to the hydroxyl group of the substrate at O- and N- position [24]. Hence, GT1s are also known as uridine diphosphate (UDP)-glycosyltransferases (UGTs) and are one of the 114 superfamilies of glycosyltransferases identified in the CAZy database (http://www.cazy.org, accessed on 26 June 2021)). The GT1s contain a unique 44-amino-acid plant secondary product glycosyltransferases (PSPG) motif near the C-terminus that is conserved across different plant species [25,26]. The PSPG box motifs are soluble enzymes and are essential for recognising the acceptors [27]. The PSPGs play crucial roles in the metabolism of endobiotics and xenobiotics in plants, and their functions are essentially parallel with those of vertebrate glucuronosyltransferase (UGATs) [28]. PSPG-catalysed glycosylation enhances the solubility of secondary metabolites and allows their storage within vacuoles, thus maintaining the metabolic homeostasis of host plants [29]. Thus, enzymatic glycosylation by these family GT1 members confers greater water solubility on the substrate, facilitating product accumulation in vacuoles. Though cytokinin glycosides are still poorly understood, a cytokinin glycosyltransferase, *UGT76C2* regulating the functions of cytokinins by its glycosylation, has been reported in *Arabidopsis thalaiana* [30]. Glycosyltransferases also play a crucial role in the inactivation and storage of SA and N-hydroxy-pipecolic acid (NHP), the two main regulators of plant responses to pathogens [30,31]. Recently, a glycosyltransferase gene (*Os6*) was cloned and overexpressed in Arabidopsis, and the purified active enzyme protein was demonstrated to be a glycosylate cytokinin [23]. Cytokinin glycosylation by glycosyltransferases fine-tunes cytokinin synthesis, metabolism, and function, which affects the transport and distribution of cytokinins in cells and tissues, associated signal transduction processes and upstream regulatory factors, and normal growth and development of plants. Hence, CGTs have been studied to understand the metabolic regulation of cytokinins and their physiological effects on plants. In contrast, the role of CGTs in the interactions of the host plants with pathogenic microbes has seldom been reported. In a high-resolution rice genetic mapping, the locus *Rsn1* of rice, regulating tissue necrosis, [32] predicted two CGT genes, *LOC_Os07g30610.1* and *LOC_Os07g30620.1,* as the potential candidates favouring susceptibility by interacting with the host-specific phytotoxic metabolite of *R. solani* anastomosis group 1A (RS AG1-IA).

Understanding the role of CGTs in biotic stress and the growth and development of rice is of great significance for understanding cytokinin-mediated immunity or susceptibility. Here, we report the genome-wide identification and in silico characterisation of rice CGTs in order to better understand their diversity. We validated the differentially expressed, unique CGT genes in disease development during infection of susceptible rice cultivars. This study will enhance our knowledge of CGT function in rice interactions with potential pathogens and will be useful to help understand the role of cytokinins in rice defence mechanisms.

## 2. Results

### 2.1. Genome Identification and Characterisation of CGT Genes

**Figure 1:**
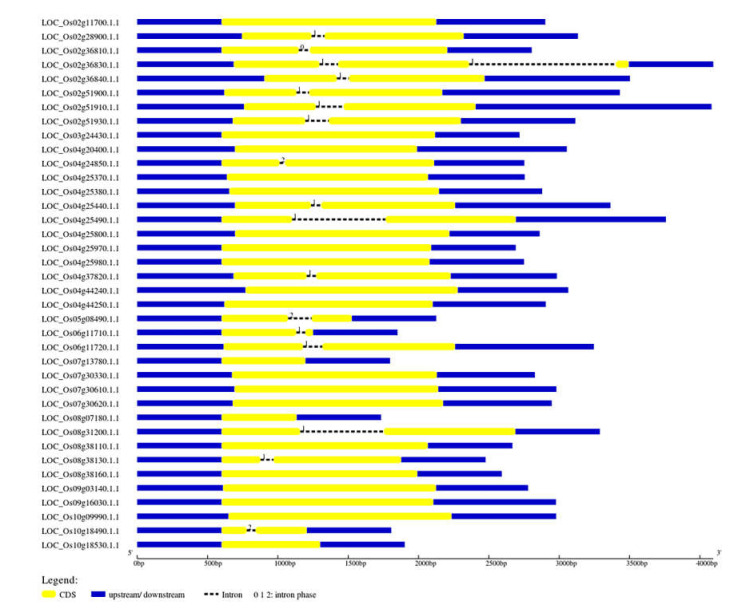
Exon/intron organization of cytokinin-O-glucosyltransferase (CGTs) genes. Yellow boxes represent exons, and black dashed lines with the same length represent introns. The upstream/downstream regions of CGT genes are indicated in blue boxes. The numbers 0, 1, and 2 represent the splicing phase of the intron. The length of exons can be inferred by the scale at the bottom.

**Figure 2:**
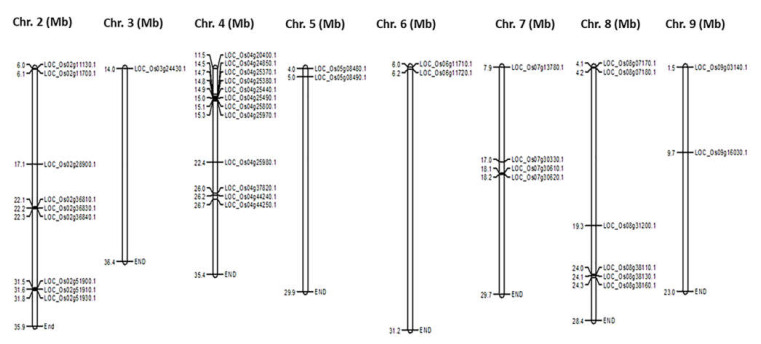
Graphical representation of physical locations of the cytokinin-O-glucosyltransferase (CGT) genes on rice chromosomes 2–10. Only tandem-duplicated genes on a particular chromosome are indicated in clusters. The chromosomal distances are given in Mbp.

In the in silico analyses, 41 CGT genes with the PP001 conserved domain were identified in rice genomic data based on BLASTP searches and other available bioinformatics tools (Table 1). The analysis of the gene structure of the CGTs indicated 0 to 12 introns in 41 genes. The presence of introns was observed in all the *CGT* sequences with the exceptions of *LOC_Os04g25370.1,* *LOC_Os08g07180.1,* *LOC_Os07g13780.1,* *LOC_Os10g18530.1,* *LOC_Os08g38160.1,* *LOC_Os08g38110.1*, and *LOC_Os03g24430.1* (Figure 1). The distribution of the 41 CGT genes on rice chromosomes was further investigated using the MSU Rice Genome Annotation Project Release (http:/rice.plantbiology.msu.edu/) database. Analysis of the chromosomal location of CGTs showed that the genes (2–12 nos) are distributed on 9 chromosomes. As shown in the graph (Figure 2), drawn using the Map Draw tool, the maximum number (12 nos) of genes is distributed on chromosome 4, whereas only a single CGT gene (*LOC_Os03g24430.1*) is located on chromosome 3 (*LOC_Os03g24430.1*).

### 2.2. Physicochemical Characterisation of Rice CGTs

The rice CGT physicochemical properties, including pI, GRAVY, EC, AI, and II were itemised (Table 1). The molecular weights of CGTs ranged from 59.7 kDa (*LOC_Os02g36830.*1) to 19.6 kDa (*LOC_Os10g18490.1*), with 544 and 180 amino acids, respectively. Though a majority (36) of the CGTs were analysed to be acidic (<7 pI) proteins, a few of them, *LOC_Os06g11710.1,* *LOC_Os07g13780.1,* *LOC_Os08g07170.1,* *LOC_Os08g07180.1,* and *LOC_Os08g38110.1,* displayed basic properties. The dipeptide-composition-based instability index (II) of the proteins ranged from 26.16 (*LOC_Os08g07180.1*) to 87.16 (*LOC_Os10g18530.1).* A large number (29) of the proteins were identified to be unstable (displaying an instability index >40), whereas 13 CGT proteins are stable (instability index <40). The highest aliphatic index of 104.43 was observed for *LOC_Os07g13780.1*, whereas the lowest aliphatic index (78.24) was recorded in *LOC_Os04g37820.1*. Though negative GRAVY values were obtained for most (58%) CGT proteins, 43% showed positive GRAVY values. In predicting their subcellular localisation in the rice plant by the integrative predictor, the CGTs were identified to be mostly located in plastids (16) followed by the plasma membrane (13), mitochondria (5), and cytosol (4). Three CGTs, *LOC_Os04g44250.1, LOC_Os08g38130.1*, and *LOC_Os10g18530.1,* are located in the extracellular membrane (Table 1).

### 2.3. Phylogenetic Analyses of CGTs

**Figure 3:**
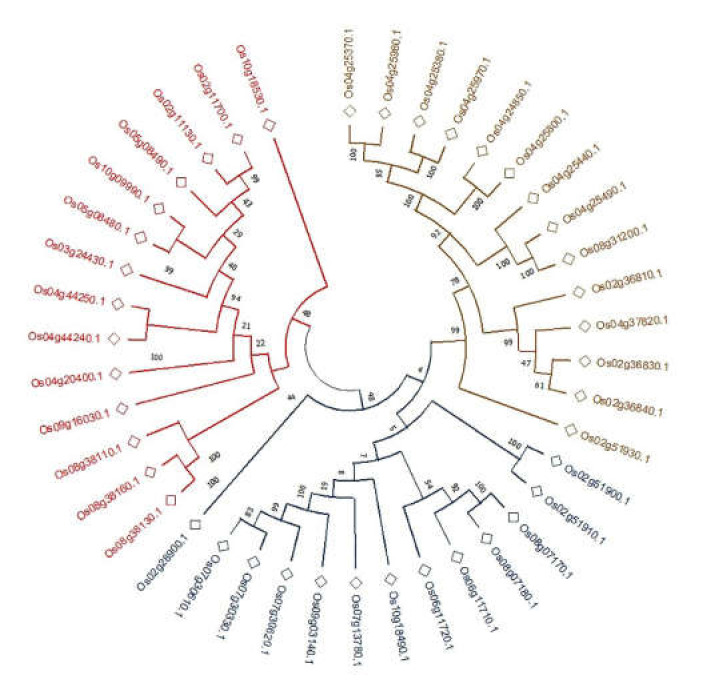
The phylogenetic tree of cytokinin-O-glucosyltransferase (CGT) genes belonging to family 1 was inferred using the neighbour-joining method. The bootstrap consensus tree inferred from 1000 replicates represents the evolutionary history of the taxa analysed. Branches corresponding to partitions reproduced in fewer than 50% of bootstrap replicates are collapsed. The percentage of replicate trees in which the associated taxa clustered together in the bootstrap test (1000 replicates) are shown next to the branches. The evolutionary distances were computed using the Poisson correction method and are in the units of number of amino acid substitutions per site. This analysis involved 41 amino acid sequences. All ambiguous positions (655) were removed for each sequence pair (pairwise deletion option). Evolutionary analyses were conducted in MEGA X.

The protein sequences of all 41 CGTs were used to delineate their evolutionary relationships. The CGTs exhibited similar evolutionary relationships for displaying homology with 70–100% bootstrap values (Figure 2). The amino acid sequences of the CGTs aligned themselves into two significant/major clusters. The first main cluster (coloured blue) showed the highest number of proteins, grouped into two sub-clusters, while the second main cluster had few members. A high level of sequence similarity (90–100%) or bootstrap supports was mainly observed for the nine gene sequences aligned in the first cluster: *LOC_Os04g25370.1,* *LOC_Os04g25980.1,* *LOC_Os04g25380.1,* *LOC_Os04g25970.1,* *LOC_Os04g24850.1,* *LOC_Os04g25800.1,* *LOC_Os04g25440.1,* *LOC_Os04g25490.1,* and *LOC_Os08g31200*. The neighbour-joining method defined the 41 CGTs into 655 positions, and Poisson correction was used to compute CGTs’ evolutionary distances (Figure 3).

### 2.4. In Silico Identification of Differential Expression of CGT Genes in Rice during Growth and Pathogen Infection

**Figure 4:**
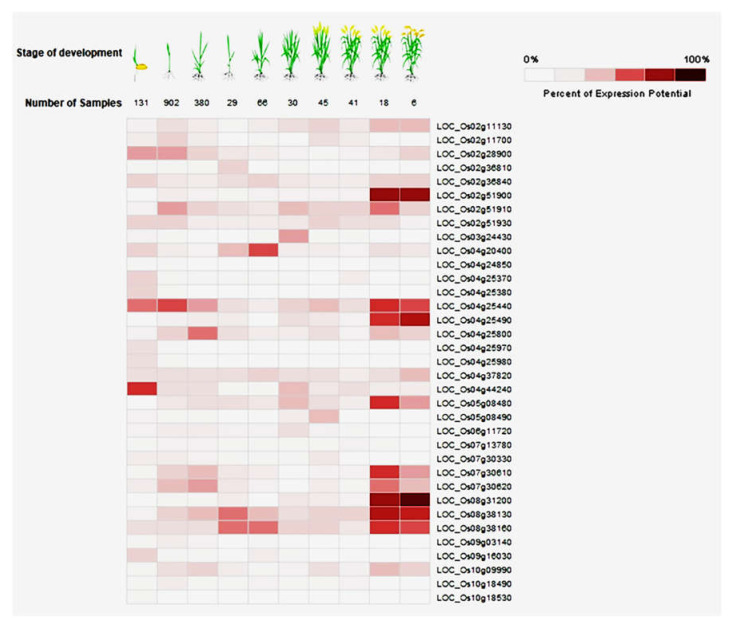
In silico (Genevestigator) deduction of differential expressions of up- and downregulated cytokinin-O-glucosyltransferase (CGT) genes of rice during different developmental stages. Downregulation is indicated by white colour and up-regulation by brown colour.

The expression of the 41 CGTs were deduced in silico using the Genevestigator tool, which contains the transcriptomics data of rice. In evaluating 1648 samples representing different developmental stages of rice, the heatmap analysis revealed a high level of expression of 16 genes during the reproductive growth stages (heading, grain filling, and maturity) of the crop (Figure 4): *LOC_Os02g11130.1,* *LOC_Os02g28900.1,* *LOC_Os02g36840.1,* *LOC_Os02g51900.1,* *LOC_Os02g51910.1,* *LOC_Os04g25440.1,* *LOC_Os04g25490.1,* *LOC_Os04g25800.1,* *LOC_Os04g37820.1,* *LOC_Os05g08480.1,* *LOC_Os07g30610.1,* *LOC_Os07g30620.1,* *LOC_Os08g31200.1,* *LOC_Os08g38130.1,* *LOC_Os08g38160.1,* and *LOC_Os10g09990.1*. In contrast, the expression of *LOC_Os04g25440.1* was higher in the vegetative stage (ranging from seedling to grain maturation) (Figure 4).

**Figure 5:**
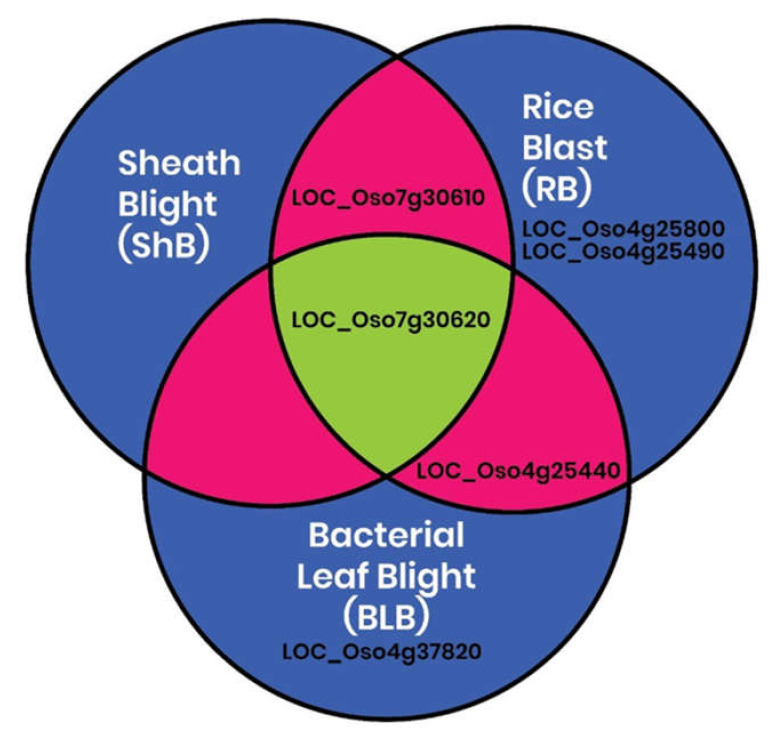
In silico prediction of 6 (out of 41) upregulated rice cytokinin-O-glucosyltransferase genes specific to sheath blight (RSB) and rice blast (RB bacterial leaf blight (BLB).

CGT expression was also evaluated among 858 samples derived from the interactions of the rice plant with various pathogens, including RS, Mor, and Xoo. The expression of all 41 genes were observed in the Genevestigator tool (Supplementary Figure S1), which was further confirmed by RiceMetaSysB (http://14.139.229.201/RiceMetaSysB, accessed on 26 April 2020)), a database of 15,135 transcriptomes generated from 241 rice samples infected with Mor and 7475 transcriptomes generated from 186 rice samples infected with Xoo [31]. RiceMetaSysB also confirmed specific upregulation of six genes in the rice Mor and Xoo pathosystems (Figure 5): *LOC_Os07g30610.1,* *LOC_Os04g25440.1,* *LOC_Os07g30620.1,* *LOC_Os04g25490.1,* *LOC_Os04g37820.1,* and *LOC_Os04g25800.1*.

### 2.5. Validation of the Expression of the In Silico Regulated CGT Genes by qRT-PCR

**Figure 6:**
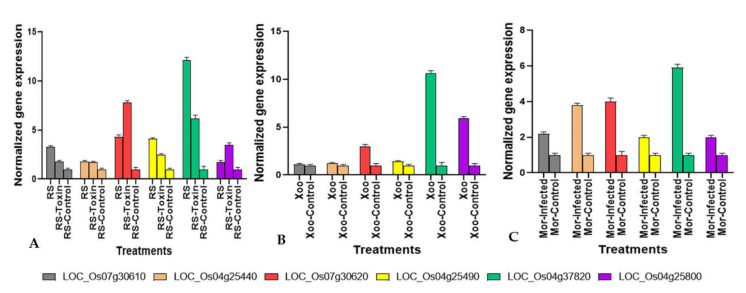
Quantification (qRT-PCR) of the expression of potential cytokinin-O-glucosyltransferase (CGT) genes of rice cultivars PBI infected with (**A**) *Rhizoctonia solani* (RS) and (**B**) *Xanthomonas oryzae* pv. *oryzae* (Xoo) and HP2216 infected (**C**) *Magnaporthe oryzae* (Mor) along with control inoculation at 48 h post-inoculation. The grey, brown, red, yellow, green, and purple bars represent the relative fold change of *LOC_Os07g30610, LOC_Os04g25440, LOC_Os07g30620, LOC_Os04g25490, LOC_Os04g37820,* and *LOC_Os04g25800,* respectively. Standard error bars show the standard deviations of three replications.

To identify the roles of the six candidate CGT genes (*LOC_*O*s07g30610.1,* *LOC_Os04g25440.1,* *LOC_Os07g30620.1,* *LOC_Os04g25490.1,* *LOC_Os04g37820.1*, and *LOC_Os04g25800*) in rice interactions with RS, Mor, and Xoo pathogens, qRT-PCR analysis of the susceptible rice varieties infected with each of the pathogens separately was conducted. At 24 h post-infection, in comparison to the SDW control, only two genes, *LOC_Os04g25800.1* and *LOC_Os07g30620.1,* showed significant levels of expression in the inoculated rice tissue of the four treatments. Among the genes, *LOC_Os04g25820.1* exhibited the highest expression, with a 5.9- to 12.1-fold increase in the inoculated rice tissue. In the rice tissue inoculated with the sclerotia and phytotoxin of RS, expression was increased by 12.1- and 6.2-fold, respectively, whereas the increase was 6- and 10.6-fold in the tissue infected with Mor and Xoo, respectively (Figure 6A). In the inoculated rice tissue, expression of the other gene, *LOC_Os07g30620.1*, increased in a range from 8- to 4-fold. The gene displayed the maximum expression of 7.8- and 4.3-fold over the control in the phytotoxin- and sclerotia-inoculated rice tissues, respectively. The rice tissues inoculated with Mor and Xoo showed 4- and 3-fold increase in expression, respectively (Figure 6A–C). Contrary to *LOC_Os04g25820.1* and *LOC_Os07g30620.*1, the expression of the genes *LOC_Os04g25440, LOC_Os04g25800,* *LOC_Os07g30610.1*, and *LOC_Os04g25490* in the rice tissue over the control were observed to be pathogen-specific. In comparison to the control treatment, significant expressions of *LOC_Os04g25440* and *LOC_Os04g25800* were observed only in the Mor (3.8-fold) and Xoo (5.9-fold) treatments, respectively (Figure 6B, C), whereas the CGT genes *LOC_Os07g30610.1* (3.3-fold) and *LOC_Os04g25490* (4.1-fold) were observed to be highly expressed versus the control in rice samples treated with RS (Figure 6A).

## 3. Discussion

The glycosylation reaction mediated by glycosyltransferases (GTs) is a significant post-translation modification affecting several cellular processes and metabolic pathways in plants, ranging from protein trafficking, molecular trafficking, cellular localisation, and cell–cell adhesion to host–pathogen interactions [33]. Among the GTs, GT1 plays a vital role in regulating the growth and development of plants and additionally modulates their responses to biotic and abiotic stresses by acting on several substrates, such as terpenes, flavonoids auxin, cytokinin, salicylic acid, etc. [34]. Previous identification methods via genetic and biochemical approaches have usually been tricky and slow; these shortcomings are recognised as primary constraints in their use for identification [35]. Progress in genomics, especially with the advancement of bioinformatics, has enabled the sequencing of several organisms, making possible comprehensive genomic identification of genes and their families within an organism [36]. The large number of GT gene sequences available in the CAZy database depicts the progress made in the study of GTs. In 2008, when there were only 90 GT families in the CAZy data base, [37] identified 609 GTs in rice. Presently, the CAZy database (http://www.cazy.org (accessed on 26 June 2021)) classifies GTs into 114 families.

In rice, the availability of the complete genomic information in the Rice Genome Annotation Project (http://rice.plantbiology.msu.edu (accessed on 30 June 2021)) provides an opportunity to investigate the diversity of these essential enzymes in greater detail. We leveraged this resource for genome-wide analysis of the UGT sub-family of GTs in rice. The study identified 41 CGTs mapped in nine out of the 12 chromosomes in rice. All the identified CGTs possess the PSPG motif of the 44-amino-acid consensus sequence, a characteristic of plant UGTs [38]. Though a single CGT gene (*LOC_Os03g24430.1*) is located on chromosome 3, the majority (12 nos) of genes are distributed on chromosome 4. This is contrary to the distribution patterns reported earlier in Arabidopsis [37], wheat [39], and cotton [40] genomes. Among the 41 CGTs, except for six genes distributed across nine chromosomes, a maximum of 12 introns are present in three CGTs. Though introns do not encode proteins, the loss or gain of introns and their insertion position in relation to the protein sequences are key clues to the evolution or diversification of the gene family [41].

In the in silico physico–chemical characterisation of the CGTs, though the CGTs belong to the same sub-family, the CGTs displayed varying molecular weights, ranging from 19.6 kDa through 59.7 kDa. Further, the CGTs were mostly acidic proteins (87.8%), which could be due to their lower isoelectric points [42]. For a protein with many basic amino acids, the isoelectric point will be high, while for an acidic protein, the pI will be lower [43]. The proteins are mostly unstable (65.85%) due to their high instability indices (>40). A protein with an instability index of <40 is predicted as stable, otherwise it is classified as unstable [44]. The proteins are determined to be predominantly hydrophilic (58.6%) due to exhibiting negative GRAVY values. Negative GRAVY score values indicate hydrophilic peptide sequences, whereas positive GRAVY scores indicate hydrophobic peptide sequences [45]. In predicting the subcellular localisation of the CGTs in the rice plant, the enzymes were distributed in the plastids (16), plasma membrane (13), mitochondria (5), and cytosol (4). Earlier, in an in silico analysis, [42] deduced a similar cellular localisation pattern of the UGTs in wheat (*Triticum aestivum* L.). Plastids are DNA-containing organelles unique to plant cells; they are directly engaged in many plant metabolic processes and contain high amounts of CK glucosides [46]. The concentration of CGT proteins in plastids gives insight into their roles in plant homeostasis, biotic and abiotic stress response, and development. Aside from synthesising many classes of molecules, plastids are plant storage units [47], and CK-O-glucosides have been reported to accumulate in rice plastids [48]. This agrees with the previous report that UGTs are predominantly located in plant intracellular fluid, from where they regulate plant hormones, such as cytokinins [49]. Plastid gene expression also plays an essential role in embryogenesis and postembryonic development; among 339 nonredundant Arabidopsis genes required for proper embryo formation, 108 encode plastid-targeted proteins [50]. Brenner et al. [51] identified five rapidly CK-induced plastid transcripts in Arabidopsis seedlings by genome-wide expression profiling, indicating a fast transfer of the CK signal to plastids or its direct perception. The CK effect on gene expression may be mediated via hormone interaction with specific proteins, and endogenous CK occurrence in plastids has been proven [48]. The importance of CKs for plastid development and function may be deduced from the partial localization of the CK biosynthetic pathway to this compartment [52].

In the phylogenetic analysis of the CGTs, the evolutionary relatedness ranged from 70–100%, and the protein sequences aligned themselves into two major clusters. Phylogenetic trees display genes in groups based on sequence similarity and are particularly valuable when studying large gene families [53]. Though nine genes on chromosome 4 exhibited a maximum genetic relatedness of 100%, fewer sequence similarities (70–100%) were observed in general among the 41 CGTs, indicating their diverse roles in plants, including detoxification of exogenous substances, cell wall synthesis, hormone modification, glycosidic bond formation, secondary metabolite synthesis, and metabolic regulation [12,37]. The decreased sequence similarities of CGTs may be due to tandem and segmental duplication events of the chromosomes [40]. Previous studies on the class III peroxidase multigenic family in rice indicate multiple gene duplication events with conservation of the amino acid sequences during evolution [54]. As suggested in previous studies, multiple copies of very similar CGT genes within chromosome 4 might have evolved to adapt to various environmental conditions. We present a hypothesis here that a proxy for the divergence of function in CGTs could be due to variations in primary sequences.

Cytokinins are known for cell division/cell differentiation regulating growth and development [11], and CGTs play a significant role in regulating their contents [12] by inactivation via glucosylation. Hence, to validate the diverse biological functions deduced for the UGTs in the in silico analyses, the expressions of the 41 CGTs were evaluated at different growth stages of rice and during biotic stress. The evaluation was made in silico with the Genevestigator tool, which contains rice transcriptomics data generated at different growth stages and during interactions with various pathogens. In the heatmap analyses of the samples representing different growth stages, i.e., seedling to grain maturity, only *LOC_Os04g25440.1* was significantly expressed at the vegetative stage, whereas 16 other CGT genes were highly expressed at the reproductive growth stage. The significance of cytokinins on inflorescence and panicle development has been well established through mutations of the cytokinin biosynthesis gene *LOG* [55] or the cytokinin degrading gene *OsCKX2* [56] of rice. Also, high expression of cytokinin biosynthesis genes during panicle development were deduced by earlier transcriptomics analysis [57,58].

Besides regulating growth and development in plants, cytokinins are also attributed with abiotic and biotic stress tolerance. In biotic stress tolerance, though cytokinin-induced susceptibility is widely known for being a pathogen-driven process in biotrophic and hemibiotrophic tumour and non-tumour forming pathogens [14,15,16,17,18,19], plant-driven cytokinin production promote resistance against various pathogens, including necrotrophic pathogens. In tomatoes, cytokinins induce systemic immunity against *Botrytis cinerea* and *Oidium neolycopersici* via a SA- and ET-dependent mechanism [59]. However, the molecular mechanisms of how plant- and pathogen-derived cytokinins oppositely affect the plant defence response have remained elusive [60]. Nevertheless, by deducing the role of cytokinins in host defence responses, it was shown that cytokinins were involved in crosstalk between the jasmonic acid/ethylene (JA/ET) and salicylic acid (SA) resistance pathways [61]. In the crosstalk, the cytokinins positively influenced the JA/ET pathway to inhibit cell death and mediated resistance to necrotrophs, whereas this effect was reversed via the SA pathway as negative influence on cytokinins caused susceptibility to necrotrophs. Cytokinin-induced immunity in plants is known to be countered by pathogens via triggering the expression of CGTs [32]. Recently, in identifying genes frequently responsive to Xoo and Mor infections in rice, ref. [62] observed that the cytokinin-related processes were most frequently repressed by the pathogens.

Amongst the genes, specific upregulation of six genes was confirmed for the rice-Mor and rice-Xoo pathosystems by the RiceMetaSysB database: *LOC_Os07g30610.1,* *LOC_Os04g25440.1,* *LOC_Os07g30620.1,* *LOC_Os04g25490.1,* *LOC_Os04g37820.1,* and *LOC_Os04g25800.1*. In validating the expression of the six candidate genes in the sheath tissue sampled at the maximum tillering stage of rice varieties susceptible to the RS, Mor, and Xoo pathogens, qPCR assays revealed that, in comparison to the SDW control, at 24 h post-infection only two genes, *LOC_Os07g30620* and *LOC_Os04g25820*, displayed significant upregulations in response to all three pathogens. The expression of *LOC_Os07g30610,* LOC*_Os04g25440, LOC_Os04g25490*, and *LOC_Os04g25800* in the rice tissue were observed to be pathogen-specific. Compared to the control, significant expression of *LOC_Os04g25440* was observed only in the Mor (3.8-fold) treatment, whereas *LOC_Os04g25800* was significantly expressed only in the rice tissues inoculated with *Xoo* (displaying a 5.9-fold higher expression over the control). The CGT genes *LOC_Os07g30610* (3.3-fold) and *LOC_*Os*04g25490* (4.1-fold) were highly expressed in rice samples treated with RS. Previously, ref. [63] predicted two unlinked loci conferring sensitivity of rice to the phytotoxin of RS for regulating necrosis and tissue chlorosis. In a high-resolution genetic map of the locus regulating tissue necrosis designated as *Rsn1*, for “*Rhizoctonia solani* necrosis gene number one”, ref. [32] predicted two CGT genes—*LOC_Os07g30610* and *LOC_Os07g30620*—of near-identical size (1449 and 1497 bp, respectively) as potential candidates of *Rsn1*. In the present study, among the *LOC_Os07g30610* and *LOC_Os07g30620* genes, only *LOC_Os07g30610* was observed to be specific to RS, whereas *LOC_Os07g30620* was induced by all three pathogens.

Among the rice pathogens used in this study, Xoo is recognised as a biotroph, whereas Mor and RS are classified as hemibiotrophic and necrotrophic fungal pathogens, respectively [64]. The significant expression of the CGT genes in rice on inoculations with either of the three pathogens exhibited varying modes of nutrition, indicating that CGTs play a significant role in alleviating cytokinin-induced immunity and can be speculated as potential susceptibility genes. Breeding for disease resistance in rice is largely constrained by the absence of donor cultivars or lines. In such cases, the candidate susceptibility genes could serve as potential alternatives to confer recessive resistance to the crop. Though the mechanisms of action of the CGTs on the cytokinins are unknown, we speculate that the glycosylation of cytokinins might have inactivated the hormone to facilitate Xoo and *M. ozyzae* infections or reversed its usual function to induce cell death in case of RS. In evaluating the mechanism of action of CGTs, ref. [65] observed that the enzymes recognise the cytokinins as acceptor molecules to form O-glucosides, which are speculated to play a role in hormone homeostasis. Later, ref. [66] observed that the enzymes also transfer the sugar group from a donor molecule that does not require a free sugar for the acceptor, and the cytokinin ribosides are shown to cause apoptosis in order to facilitate direct degradation of the carbohydrate donor [65,67].

The forgoing studies conclude that the candidate CGT genes deduced from the in silico and in planta analyses have the potential for significant expression during different growth stages and with inoculation of different pathogens in the rice crop. In the latter, the genes serve as potential susceptibility genes for facilitating pathogen infection. Cloning and functional analysis of these genes may enable a better understanding of the molecular mechanisms of cytokinin-induced defence responses in the host. Besides, altering these genes will directly affect their interactions with the pathogen’s effector and genetically lead to recessive resistance.

## 4. Materials and Methods

### 4.1. Genome-Wide Identification and Characterisation of Rice CGT Genes

The compressive identification of the CGT genes was made using three complementary methods. First, 609 UGT sequences of rice were obtained (https://ricephylogenomics.ucdavis.edu/cellwalls/gt/index.shtml (accessed on 13 April 2020)). BioMart Ensemble plants (https://plants.ensembl. org/index.html (accessed on 13 April 2020)) and Uniprot (http://www.uniprot.org/ (accessed on 13 April 2020)) were used to obtain genomic, transcriptomic, proteomic, and annotated data of the retrieved sequences. Next, using the conserved UDPGT (PF00201.17) domain, the sequences were screened using the Hidden Markov Model (HMMER) (http://www.ebi.ac.uk/Tools/hmmer/ (accessed on 13 April 2020)) and the PFAM program (http://pfam.xfam.org/ (accessed on 13 April 2020)) at *p* < 0.001. Finally, the PSPG box 44-amino-acid was used as a query to screen the CGT genes via a local BLASTP search with significant cut-off E-values of 0.01 and 0.03 for sequence and hit, respectively. Because the box 44-amino-acids are characteristic of the CG family, proteins without or containing partial PSPG boxes were removed. Finally, after careful and complete curation, 41 protein genes were validated in the rice genome. The identification of signal peptides in UGT sequences was performed by SignalP 4.1 Server (version 4.1). The structure of the CGT genes was determined using the server (http://gsds.gao-lab.org/ (accessed on 30 April 2020)) to identify introns and exons. The intron phases were determined as follows: introns positioned between two codons were defined as phase 0, introns positioned between the first and second base of codon were defined as phase 1, and introns positioned between the second and third base were defined as phase 2 [68]. Each of the CGT sequences was searched against the genomic data available in MSU Rice Genome Annotation Project Release (http:/rice.plantbiology.msu.edu/ (accessed on 30 April 2020)) using the default settings of the database to detect their CDS coordinates (5′–3′). The genes were individually plotted onto the 12 rice chromosomes from the short-arm telomere to the long-arm telomere, as per their increasing physical locations (Mbp), and, subsequently, their physical locations were depicted with MapChart software (version 2.2) [69]. 

### 4.2. Physicochemical Characterisation of CGTs

The ExPasy website (http://web.expasy.org/protparam/ (accessed on 15 May 2020)) tool was employed to determine the physicochemical properties of the CGT proteins, including grand average of hydropathicity (GRAVY), instability index (II), protein lengths, molecular weights (MW), theoretical isoelectric points (pI), extinction coefficient (EC), and aliphatic index (AI). The subcellular localisation of the proteins was predicted at a *p*-value of <0.05 using an integrative subcellular localisation predictor for plants, which has 11 prediction tools (PSI) (http://bis.zju.edu.cn/psi/ (accessed on 15 May 2020)) [70].

### 4.3. Phylogenetic Analysis of CGTs

The protein sequences of CGT were matched with BLOSUM using ClustalX system as the protein weight matrix. COBALT and MUSCLE (http://www.ebi.ac.uk/Tools/msa/muscle/ (accessed on 19 May 2020)) tools were used to conduct the alignments of the rice CGT protein sequences. Evolutionary distances of the protein sequences and phylogenetic trees were built with the neighbour-joining (NJ) method of the MEGA X program (http://www.megasoftware.net/ (accessed on 25 May 2020)). The tree reliability was tested using a Poisson correction, and phylogenetic tree images were drawn using MEGA X (Version 10) with 1000 replicate bootstrapping.

### 4.4. In Silico Expression Analysis of CGTs

The expressions of the CGT genes deduced in silico were identified from the RNA-seq data of rice tissues sampled at different growth stages and subjected to biotic stresses using the GENEVESTIGATOR^®^ tool (https://genevestigator.com/gv/ accessed on 28 May 2020)). The common responsive genes were confirmed using RiceMetaSysB (http://14.139.229.201/RiceMetaSysB/ accessed on 28 May 2020)) [71]. Expression of all the CGT genes at different developmental stages and during different biotic stress responses was analysed by Genevestigator [72] by selecting development and perturbations, respectively, in the search tool. Next, the 41 CGT genes were used as queries in the “data input” section. Adjusted *p*-value (false discovery rate) <0.05 and minimal log2 (fold change) = 2 were selected as criteria in the selection of differentially expressed genes [72]. Both microarray and RNA-seq databases were explored for expression analysis. Besides Genevestigator, expression analysis of these genes was also carried out by exploring the RiceMetaSysB database for biotic stresses [71,72]

### 4.5. Validation of the Expression of the In Silico Regulated CGT Genes

Seeds of rice cultivars, Pusa Basmati 1 (PB1) (susceptible to ShB and BLB [33,34]) and HP2216 (susceptible to blast [45]) were obtained from the Division of Genetics, IARI, New Delhi, surface-sterilised with 1% sodium hypochlorite and sown separately in three plastic pots (size 7” × 7”) containing wetland soil. The seedlings were grown in a greenhouse at the National Phytotron Facility of the host institute at 80% relative humidity and 28 °C with 16/8 h of day/night and 250 µmol light intensity. At 30 days after sowing, the seedlings of each of the cultivars were transplanted in three plastic pots (size 7” × 7”), and the 45-day-old seedlings, each constituting five sheaths, were used for the experiments.

Inoculation of PB1 was carried out with sclerotia and phytotoxic metabolite of a highly virulent *R. solani* AG1 strain, RIRS-K (ITCC No-7479). Three sclerotia from a pure culture (7 days old) of the fungus were inoculated onto the rice sheaths, and the inoculated sheaths were wrapped with parafilm strips for proper sclerotia attachment. The host-specific phytotoxin was extracted from 1 L cell-free culture filtrate prepared from Richard’s broth inoculated with fungal mycelium as described earlier [33]. Briefly, the culture filtrate was dehydrated using ethyl acetate in a rotary evaporator at 43 °C. The dried crude extract was then purified by column chromatography using a mixture of chloroform and methanol as a mobile phase. The purified phytotoxin (50 µL of 1000 ppm conc.) was slowly infiltrated into pinprick injuries on the sheaths. In another experiment, inoculation of the HP2216 cultivar was made at the panicle-formation stage with a conidial suspension (1 × 10^5^ conidia/mL) of Mor strain Mo-ni-0025 as previously described [45]. To prepare the conidial suspension, a 5 mm mycelial disc of the fungus was inoculated into 100 mL of potato dextrose broth and incubated for 10 days on a rotary shaker at 28 ± 2 °C. The mycelium was then removed by passing the broth through a muslin cloth. Inoculation of the PB1 cultivar with the Xoo inoculum was prepared by suspending the cells of the bacterial strain, ITCC-BB0003 maintained on peptone sucrose agar (PSA) solid media (1 L) (peptone-10 g, sucrose-10 g, L-glutamatic acid-1 g, agar 20 g,) at 28 °C for 24–48 h in 10 mM sterilised MgCl_2_ solution and diluting the suspension to 1 × 10^6^ colony-forming units (cfu) mL^–1^. Inoculation was made on leaf blades of the cultivar using scissor tips dipped in the suspension by a leaf clipping technique described earlier [34]. Uninoculated plant samples were used as controls in all three experiments. The infected tissues of two biological samples of each experiment were collected at 24 h post-inoculation, washed in running tap water, and homogenised with liquid nitrogen in a pre-chilled mortar and pestle before storing at −80 °C.

Total RNA was isolated from the ground tissues of each of the three experiments using TRIzol Reagent (Thermo) following the manufacture’s protocol. The quality of isolated RNA was ascertained by measuring the concentration using NanoDrop 2000 (Thermo). The RNA was DNase treated to remove any traces of DNA contamination, followed by RNA purification. Superscript III first-strand cDNA synthesis (Invitrogen) was used to synthesise cDNA from the RNA. The synthesised cDNA was subjected to qRT-PCR analysis to validate the expression of six candidate CGT genes identified in silico: *LOC_Os07g30610.1,* *LOC_Os04g25440.1,* *LOC_Os07g30620.1,* *LOC_Os04g25490.1,* *LOC_Os04g37820.1,* and *LOC_Os04g25800*. Primers for the qRT-PCR analyses of the CGT genes were designed with the PrimerQuest (Integrated DNA Technologies) tool (Supplementary Table S1). The cDNA was normalised with actin, and a reaction mix of 30 µL was prepared with the required amount of diluted cDNA, forward and reverse primers (0.3 μL each), 6-carboxy-x-rhodamine (ROX) fluorescence dye (0.4 µL), 2X SYBR green master mix (15 µL), and nuclease-free water. The amplification was conducted in a Light Cycler^®^ 480 II (Roche) at 95 °C for 3 min, which was followed by 40 cycles of 95 °C for 10s, 60 °C for 10 s, and 72 °C for 10 s. Each reaction was run in triplicate, and values of relative fold change between calibrator and experimental samples were determined by the 2-ΔΔCt method. Melting curve analysis was employed to monitor primer-template specificity. Significant variations between the control and infected samples were calculated by two-way ANOVA and designated by the asterisk sign above the error bars (*p* < 0.05).

### 4.6. Statistical Analyses

All the experiments were replicated as described and carried out in a completely randomised design. The pot culture experiments were repeated with similar results, hence one representative trial is indicated. Statistical analyses of the experiments were performed using the package IRRISTAT version 92-1 developed by the International Rice Research Institute Biometrics Unit, the Philippines. Differences between treatments mean values were determined following the LSD test at 0.05 probability level.

## Figures and Tables

**Table 1:** List of putative cytokinin-O-glucosyltransferases genes deduced from rice genomic data.

Gene Stable ID/ Locus Name	MW (kDa)	PL	_P_I	II	AI	GRAVY	SL
LOC_Os02g11130.1	54.60	501	5.1286	35.06	90.58	0.035	Plasma membrane
LOC_Os02g11700.1	55.10	508	5.1228	39.92	84.97	−0.043	Plasma membrane
LOC_Os02g28900.1	53.30	494	4.7694	36.37	86.54	0.034	Plastid
LOC_Os02g36810.1	54.20	508	5.7206	40.99	84.13	0.006	Plasma membrane
LOC_Os02g36830.1	59.70	544	5.7414	37.71	82.27	−0.081	Plastid
LOC_Os02g36840.1	54.30	493	5.9562	40.3	80.52	−0.141	Mitochondria
LOC_Os02g51900.1	53.50	486	5.1939	43.07	82.82	−0.089	Plasma membrane
LOC_Os02g51910.1	52.80	482	5.6463	41.46	81.46	−0.091	Plastid
LOC_Os02g51930.1	53.50	485	5.8247	40.92	83.95	−0.18	Plasma membrane
LOC_Os03g24430.1	54.00	505	6.511	40.82	88.44	0.06	Plastid
LOC_Os04g20400.1	47.30	431	6.6482	58.07	84.79	−0.192	Plastid
LOC_Os04g24850.1	53.00	490	5.7159	37.36	89.84	−0.05	Plastid
LOC_Os04g25370.1	52.10	476	5.9424	38.31	86.56	−0.052	Plasma membrane
LOC_Os04g25380.1	53.90	496	6.8259	42.23	88.62	−0.033	Plastid
LOC_Os04g25440.1	54.00	497	6.1201	45.32	83.44	−0.043	Mitochondria
LOC_Os04g25490.1	51.30	475	4.8767	43.16	86.26	−0.029	Plasma membrane
LOC_Os04g25800.1	54.70	507	5.5869	37.86	89.73	−0.009	Plastid
LOC_Os04g25970.1	53.80	496	6.6935	41.24	88.24	−0.03	Plastid
LOC_Os04g25980.1	53.90	492	6.3781	40.63	86.93	−0.057	Mitochondria
LOC_Os04g37820.1	54.10	491	5.5712	44.92	78.24	−0.136	Plasma membrane
LOC_Os04g44240.1	53.90	502	6.1602	39.13	87.54	0.086	Cytosol
LOC_Os04g44250.1	53.10	493	6.857	43.54	89.88	0.096	Extracellular
LOC_Os05g08480.1	56.70	544	6.6946	44.16	92.14	0.189	Plasma membrane
LOC_Os05g08490.1	28.60	252	5.841	39.17	89.8	−0.033	Plastid
LOC_Os06g11710.1	20.80	195	9.5658	52.36	83.89	−0.088	Cytosol
LOC_Os06g11720.1	54.30	502	5.683	52.75	85.23	−0.108	Mitochondria
LOC_Os07g13780.1	20.80	198	9.0927	52.87	104.43	0.266	Plastid
LOC_Os07g30330.1	52.10	485	5.7028	40.05	94.41	0.194	Mitochondria
LOC_Os07g30610.1	51.60	482	5.2655	50.19	89.79	0.071	Plastid
LOC_Os07g30620.1	52.90	498	6.1141	46.23	89.9	0.134	Plastid
LOC_Os08g07170.1	23.20	217	7.7053	27.25	87.5	−0.065	Plasma membrane
LOC_Os08g07180.1	19.00	177	7.6701	26.17	92.22	−0.049	Cytosol
LOC_Os08g31200.1	54.10	497	5.2116	42.04	85.18	−0.038	Cytosol
LOC_Os08g38110.1	50.90	488	7.2976	49.36	88.78	0.191	Plasma membrane
LOC_Os08g38130.1	41.90	394	6.0123	41.66	89.32	0.059	Extracellular
LOC_Os08g38160.1	49.00	463	6.1379	39.73	95.82	0.238	Plastid
LOC_Os09g03140.1	54.90	504	4.8158	48.76	96.19	0.023	Plastid
LOC_Os09g16030.1	54.10	501	5.3122	34.3	91.19	0.1	Plastid
LOC_Os10g09990.1	56.30	528	4.768	32.3	92.86	0.085	Plasma membrane
LOC_Os10g18490.1	19.50	180	6.7946	45.69	90.6	−0.014	Plasma membrane
LOC_Os10g18530.1	25.80	233	6.1427	87.16	87.16	0.134	Extracellular

PL, protein length; pI, isoelectric point; MW, molecular weight; SL, subcellular location; GRAVY, grand average of hydropathy; II, instability index; AI, aliphatic index.

## Data Availability

Data is contained within the article or Supplementary Materials.

## Supplementary Materials

The following are available online at https://www.mdpi.com/article/10.3390/plants11070917/s1, Figure S1: In-silico (Genevestigator) deduced differential expressions of up- and downregulated cytokinin-Glucosyltransferase (CGT) genes of rice, Table S1: Primers used in PCR assay to assess the expressions of CGT genes in on challenge inoculation with *M. oryzae, Xoo* and *R. solani*.
