# Supplementary material for: Genome-Wide Identification and Characterisation of Cytokinin-O-Glucosyltransferase (CGT) Genes of Rice Specific to Potential Pathogens
Source: Plants (Basel). 2022 Mar 29;11(7):917. doi: 10.3390/plants11070917 (PMC9002877; doi:10.3390/plants11070917)
Supplement: Supplementary file 1 [file plants-11-00917-s001.zip › plants-1608411-supplementary.pdf]

plants

MDPI

Supplementary Material

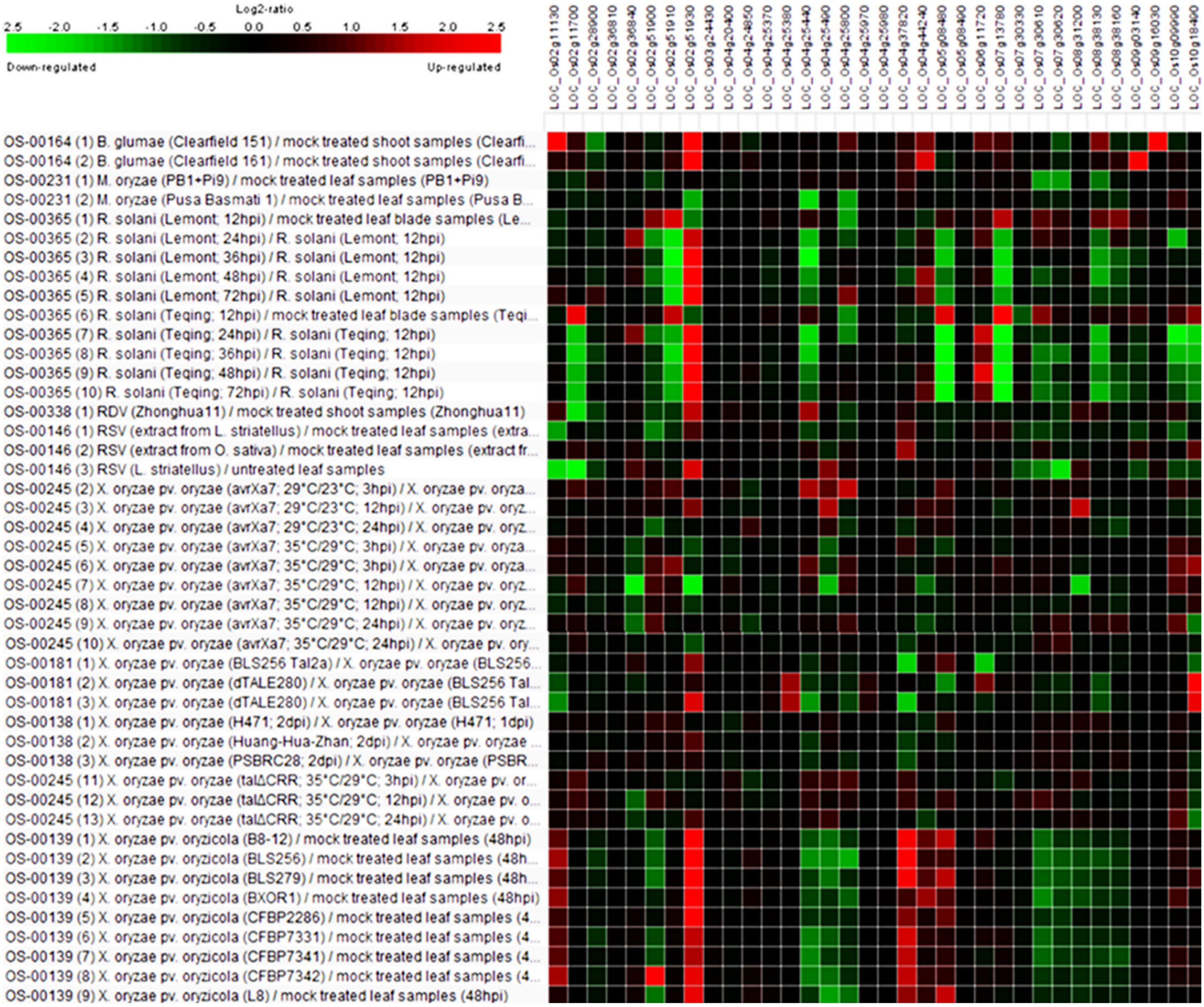

Figure S1. *In silico* (Genevestigator) deduced differential expressions of up-and downregulated cytokinin-Oglucosyltransferase (CGT) genes of rice in response to infections with *Rhizoctonia solani*, *Magnaporthe oryzae*, *Xanthomonas oryzae* pv. *oryzae*, and *Xanthomonas oryzae* pv. *oryzicola*. Downregulation is indicated by green colour and upregulation by red colour.

Table S1. Primers used in PCR assay to assess the expressions of CGT genes in on challenge inoculation with *M. oryzae*, *Xoo* and *R. solani*.

Gene	Primers	Sequence (5′-3′)
LOC_Os04g25440	CGT-F	GGACGCACACGTAACAGTAA
	CGT -R	GTCAAGTCATGGAGGCAAGT
LOC_Os04g25800	CGT -F	TGCACAAGAGTGGAAAGAGAA
	CGT -R	CTCACCATTCACCCTGCTAAT
LOC_Os04g25490	CGT -F	TGGACACGAAGATGGATTGG
	CGT-R	CGTCACGTCGATGAAGAAGT
LOC_Os04g37820	CGT -F	GACGCATACAGAGACATGGG

	CGT-R	GTTGACGAAGGTGATGTGGA
LOC_Os07g30620.1	CGT -F	CTAATCAGGCATATCTTCCCCAC
	CGT -R	TCACCCAAGTCGTTGTAGAAAG
LOC_Os07g30610.1	CGT-F	GTATCAACACCTTCCACCCC
	CGT-R	GATCTTGTACACGCCTAGAGG
